# Parental mental disorders and offspring schizotypy in middle childhood: an intergenerational record linkage study

**DOI:** 10.1007/s00127-023-02455-7

**Published:** 2023-03-13

**Authors:** Kirstie O’Hare, Kristin R. Laurens, Oliver Watkeys, Stacy Tzoumakis, Kimberlie Dean, Felicity Harris, Richard J. Linscott, Vaughan J. Carr, Melissa J. Green

**Affiliations:** 1 Discipline of Psychiatry and Mental Health, School of Clinical Medicine, University of New South Wales, Sydney, Australia; 2 School of Psychology and Counselling, Queensland University of Technology (QUT), Brisbane, Australia; 3 School of Criminology and Criminal Justice, Griffith University, Southport, Australia; 4 Griffith Criminology Institute, Mount Gravatt, Australia; 5 Justice Health and Forensic Mental Health Network, Sydney, NSW Australia; 6 Department of Psychology, University of Otago, Otago, New Zealand; 7 Neuroscience Research Australia, Sydney, Australia; 8 Department of Psychiatry, Monash University, Melbourne, Australia; 9 Discipline of Psychiatry and Mental Health, School of Clinical Medicine, UNSW Sydney, Level 1, AGSM Building, Botany Street, Sydney, NSW 2052 Australia

**Keywords:** Familial risk, Mental illness, Schizophrenia-spectrum disorders, Psychosis latent profile analysis

## Abstract

**Purpose:**

To investigate relationships between distinct schizotypy risk profiles in childhood and the full spectrum of parental mental disorders.

**Methods:**

Participants were 22,137 children drawn from the New South Wales Child Development Study, for whom profiles of risk for schizophrenia-spectrum disorders in middle childhood (age ~ 11 years) were derived in a previous study. A series of multinomial logistic regression analyses examined the likelihood of child membership in one of three schizotypy profiles (*true schizotypy*, *introverted schizotypy*, and *affective schizotypy*) relative to the children showing *no risk*, according to maternal and paternal diagnoses of seven types of mental disorders.

**Results:**

All types of parental mental disorders were associated with membership in all childhood schizotypy profiles. Children in the *true schizotypy* group were more than twice as likely as children in the *no risk* group to have a parent with any type of mental disorder (unadjusted odds ratio [OR] = 2.27, 95% confidence intervals [CI] = 2.01–2.56); those in the *affective* (OR = 1.54, 95% CI = 1.42–1.67) and *introverted schizotypy* profiles (OR = 1.39, 95% CI = 1.29–1.51) were also more likely to have been exposed to any parental mental disorder, relative to children showing *no risk*.

**Conclusion:**

Childhood schizotypy risk profiles appear not to be related specifically to familial liability for schizophrenia-spectrum disorders; this is consistent with a model where liability for psychopathology is largely general rather than specific to particular diagnostic categories.

**Supplementary Information:**

The online version contains supplementary material available at 10.1007/s00127-023-02455-7.

## Introduction

Schizotypy refers to a set of observable behavioural, cognitive, and personality characteristics that reflect liability for schizophrenia [1, 2]. The concept of schizotypy developed from observations that healthy relatives of individuals with psychosis displayed traits that were qualitatively similar to symptoms of schizophrenia [3]. This led to the hypothesis that the underlying aetiology for schizophrenia could result in a range of phenotypes, from schizotypy through to schizophrenia, and that only a minority of those with this underlying liability would go on to develop schizophrenia [1–3]. More recently, researchers have focussed on understanding subthreshold, symptom-like experiences of psychosis (e.g. psychotic-like experiences [PLEs]) as part of a spectrum of liability for psychosis and schizophrenia [4, 5]. While PLEs represent one aspect of liability for psychosis (reflected in reality distortion and other unusual experiences), schizotypy is a multifaceted construct, with ‘positive’ features (that overlap with PLEs), alongside ‘negative’ and cognitive features [6], reflecting the continuum of experience from subclinical symptomatology through to mental disorder [4], providing a developmental framework for understanding schizophrenia risk throughout the lifespan [7, 8].

The strongest known predictor for schizophrenia-spectrum disorders is having an affected first degree biological relative [9]. Individuals with a parent with schizophrenia have an eightfold increased risk of developing schizophrenia, compared to healthy controls [10]. Unaffected relatives of people with schizophrenia also have higher scores on measures of schizotypy [11–14]. Further, parental schizophrenia-spectrum disorders are associated with early childhood social-emotional dysfunction in offspring [15–18], indicating that familial liability for schizophrenia may be expressed as an observable phenotype throughout development.

While the relationship of schizotypy to familial schizophrenia-spectrum disorders has been well established, there has been limited research examining schizotypy among offspring of parents with mental disorder diagnoses other than schizophrenia. Use of population registers and/or familial high-risk studies have indicated that children of parents with mental disorder are at increased risk of a broad range of psychopathology, not limited to the specific type of disorder that their parent is diagnosed with [19–21]. Further, parental mental disorders are related not just to diagnosed mental disorder, but also to a range of early childhood vulnerability or liability to psychopathology in offspring [22]. These epidemiological findings are supported by evidence that a substantial proportion of genetic factors associated with mental disorders are shared between disorders [23–25], and that exposure to the same type of environmental risk factors (e.g. maltreatment, urbanicity, prenatal complications) are associated with increased risk of many different mental disorders [26, 27]. Intergenerational transmission of mental disorders (through genetic and environmental factors) is therefore likely to be transdiagnostic.

Only a few recent studies have explored the relationship of subthreshold psychosis symptoms with familial liability outside the schizophrenia-spectrum. One familial high-risk study found that offspring of parents with any severe mental illness (schizophrenia, bipolar disorder or major depressive disorders) were more likely than controls (i.e. children of parents with no disorder) to experience disturbances in thought, perception, and other mental processes associated with psychosis, and that likelihood did not differ across disorder classifications [28]. Offspring of parents with schizophrenia and bipolar disorder were also found to be at an increased risk of “severe” PLEs (as rated by a health professional) compared to controls [29]. Further, in the general population, delusion-like experiences (a subset of PLEs) among offspring have been associated with a range of parental mental disorders [depression, anxiety, schizophrenia, bipolar, and substance use disorders; 30]. These findings suggest that subthreshold psychosis symptoms among offspring are not exclusively related to parental schizophrenia-spectrum disorders. However, most of this research has focussed on a single (i.e. reality distortion) facet of schizotypy, and potential relationships with other parental disorders (e.g. child-onset disorders and personality disorders) have not yet been investigated.

The different components of schizotypy may not be uniformly related to familial liability for mental disorder. Previous studies have confirmed the existence of cognitive-perceptual, interpersonal, and disorganised dimensions of schizotypy [6]. However, there is little consensus on which of these features, or their particular combinations, are most strongly related to familial liability for schizophrenia-spectrum disorders [31]. A review of eight studies found that the interpersonal features of schizotypy had strong associations with familial schizophrenia, while cognitive-perceptual features had weak associations; results for disorganised features were mixed, with some studies finding strong effects and others weak effects [32]. One explanation for these differences in the relationships between dimensions of schizotypy and familial risk of schizophrenia (or other mental disorders) is that there may be subgroups of children showing different patterns of schizotypy that have distinct pathological pathways [2, 33]. That is, the association with parental history of mental disorders may not be with any individual schizotypal dimension in the population, but rather may be associated with the co-occurrence or pattern of schizotypal features *within* an individual offspring [34].

Person-centred approaches to the study of childhood risk for psychosis (and other mental disorders) can be used to identify patterns of traits within individuals. The aim of the present study was to investigate relationships of distinct (person-centred) schizotypy risk profiles in childhood with the full spectrum of parental mental health diagnoses, using data from the New South Wales-Child Development Study (NSW-CDS). Hypotheses were that (1) offspring classed in any schizotypal (schizophrenia-spectrum risk) profile would be more likely to have a parent with a diagnosed mental disorder, compared to those showing no such risk profile; and (2) all types of parental mental disorder would be associated with schizotypy group membership among offspring, but the strongest relationship would be with parental diagnosis of schizophrenia-spectrum disorder.

## Methods

### Study setting and record linkage

Data were drawn from Wave 2 of the New South Wales Child Developmental Study (NSW-CDS; http://nsw-cds.com.au/), an Australian intergenerational, longitudinal, population-based cohort study that uses record linkage to combine routinely collected administrative record data from multiple agencies with cross-sectional surveys [35]. In 2016, Wave 2 linkage was conducted for 91,635 children and their parents [36]. Record linkage was conducted by the NSW Centre for Health Record Linkage (CHeReL; http://www.cherel.org.au/) using probabilistic record linkage methods across a set of identifiers, with an estimated false positive linkage rate of < 0.5% [36]. Ethical approval was obtained from the NSW Population and Health Services Research Ethics Committee (HREC/15/CIPHS/21).

The Wave 2 linkage included 27,792 children who completed the Middle Childhood Survey (MCS), a self-report assessment of psychosocial and behavioural functioning administered to children in their final year of primary school in 2015 [37]. The MCS was administered in 829 NSW schools (35% of eligible schools) and was completed by 31.4% of year 6 children enrolled in NSW at the time.

### Participants

Participants were 22,137 children from the NSW-CDS cohort with available data on the MCS *and* linked maternal records from the NSW Ministry of Health (for years 2000–2016); parent–child data linkage was possible for children with births registered in NSW between 2000 and 2006. Of these children, 21,476 had linked data for both mothers and fathers, and 661 for their mother only. The mean age of children at the time of the MCS was 11.9 years (SD = 0.37 years).

### Measures

#### Schizotypy

Three putative profiles of schizotypy were identified via latent profile analyses of 59 items from the MCS (see [34] and Supplementary Materials). The profiles were characterised by (1) high levels of cognitive disorganisation, impulsive non-conformity, introversion, and self-other disturbance, intermediate levels of anxiety/depression, and low levels of unusual experiences, labelled ‘*true schizotypy’* (representing 5.9% of the sample), (2) high levels of unusual experiences and anxiety/depression, labelled ‘*affective schizotypy’* (20.2%), (3) high introversion, intermediate levels of cognitive disorganisation, impulsive non-conformity, self-other disturbance, but no unusual experiences or anxiety/depression, labelled ‘*introverted schizotypy’* (19.2%), and (4) the largest group of children showing no signs of schizotypy, labelled ‘*no risk’* (54.9%).

#### Parental mental disorder

Mental disorder diagnoses for mothers and fathers of the child cohort were derived from the NSW Ministry of Health’s Mental Health Ambulatory (for years 2000–2016), Admitted Patient (years 2000–2016), and Emergency Department (years 2000–2016) data collections. Specific types of mental disorder, recorded as primary or additional diagnoses, were defined by International Classification of Disease, revision 10, Australian Modification codes (ICD-10-AM; see Supplementary Materials). ICD-10-AM codes (or Systemized Nomenclature of Medicine [SNOMED] codes converted to ICD-10-AM codes) for any emergency department visit, admission to hospital or episode of ambulatory (i.e. community or outpatient) service contact up until 31 December 2016 were categorised into seven broad diagnostic categories (noting that an individual could be categorised into more than one category over their life course):

1. Schizophrenia-spectrum disorders (schizophrenia, schizoaffective disorder, other non-affective psychoses).
2. Affective psychotic disorders (bipolar disorders, depressive psychosis, postnatal affective psychosis, other affective psychoses).
3. Common mental disorders (depressive disorders, major depressive disorders, anxiety/neurotic disorders).
4. Personality disorders (cluster A, cluster B, cluster C, other/unspecified).
5. Substance abuse (intoxication, substance use disorders).
6. Other adult-onset disorders (organic disorders, eating disorders, sleep disorders, disorders not otherwise classified or not yet allocated).
7. Other childhood-onset disorders (hyperkinetic disorders, conduct disorders, other childhood emotional disorders).

As well as these seven types of mental disorder diagnoses, we derived a global index of ‘any parental mental disorder’ (not specific to any category) evident among either parent (i.e. at least one parent) as well as indicators of ‘any mental disorder’ for mothers and fathers separately.

#### Covariates

Three socio-demographic factors (child’s sex, Aboriginal and Torres Strait Islander [Indigenous] background, and socio-economic disadvantage) were considered potential confounders of the relationship between parental mental disorder and childhood schizotypy and were included as covariates in adjusted models. Each child’s sex was determined from responses on the MCS. A binary indicator of Indigenous background was determined if either the child or their parent(s) were designated as of Aboriginal and Torres Strait Islander background in any available record in the NSW-CDS collection [36]. Socio-economic disadvantage was measured using the 2011 Socio-economic Indexes for Areas, Index for Relative Socio-economic Disadvantage (SEIFA IRSD) developed by the Australian Bureau of Statistics [38] and based on the child’s home postcode collected in the MCS. A binary indicator of socio-economic disadvantage was calculated, with membership in the bottom quintile of the SEIFA IRSD considered ‘disadvantaged’.

### Analysis

A series of unadjusted and adjusted multinomial regression analyses were used to estimate associations between parental mental disorder diagnosis and offspring membership in one of the three schizotypy profiles, relative to the children showing no schizotypy (i.e. *no risk* for schizophrenia-spectrum disorders). Analyses were conducted first for “any parental mental disorder diagnosis” (i.e. at least one parent, mother *or* father, with *any* type of diagnosis), then for (*any*) maternal diagnoses and paternal diagnoses, separately. An indicator of both parents (i.e. mother *and* father) having received *any* type of psychiatric diagnosis was also investigated. Then, analyses were conducted using the seven broad disorder categories described above, first for any parent, then for maternal mental disorder diagnoses, and last for paternal mental disorder diagnoses. Analyses resulted in odds ratios (ORs) with 95% confidence intervals (CIs) as measures of effect size, with ORs of 1.00–1.49 interpreted as small, 1.50–2.49 as medium, and 2.50 or more as large [39]. Where appropriate, associations were compared in terms of the size of point estimates, and whether 95% confidence intervals were non-overlapping as a means of indicating statistically significant differences in the associations between schizotypy group membership and parental mental disorder diagnoses [40].

## Results

Descriptive statistics for the sample are presented in Table 1. Of the 22,137 children in the sample, 23.5% had a parent who had received a psychiatric diagnosis. Common mental disorders were the most prevalent, with 16.2% of children having an affected parent; 2.1% of children had a parent with a schizophrenia-spectrum disorder diagnosis; and 1.8% of children had a parent with an affective psychotic disorder diagnosis. The distribution of parental mental disorders according to child membership in one of the three schizotypy risk groups (as well as children showing no risk) is presented in Table 2.

**Table 1:** Prevalence of schizotypy group membership, parental mental disorder diagnoses, and covariates

Characteristic	Number of Children
**Total sample**	22,137
**Schizotypy group membership**	
No risk	12,080 (54.6%)
Introverted Schizotypy	4,473 (20.2%)
Affective Schizotypy	4,261 (19.2%)
True Schizotypy	1,323 (6.0%)
**Any parental mental disorder**	**Number of children with affected parent/s**
Any parent	5,199 (23.5%)
Maternal mental disorder	3,592 (16.2%)
Paternal mental disorder	2,370 (10.7%)
Both parents	763 (3.4%)
**Parental mental disorder diagnosis**	
**Schizophrenia-spectrum disorder**	
Any parent	464 (2.1%)
Maternal mental disorder	260 (1.2%)
Paternal mental disorder	217 (1.0%)
**Affective psychotic disorder**	
Any parent	398 (1.8%)
Maternal mental disorder	323 (1.5%)
Paternal mental disorder	130 (0.6%)
**Common mental disorders**	
Any parent	3,593 (16.2%)
Maternal mental disorder	2,595 (11.7%)
Paternal mental disorder	1,284 (5.8%)
**Personality disorders**	
Any parent	487 (2.2%)
Maternal mental disorder	314 (1.4%)
Paternal mental disorder	188 (0.8%)
**Substance use disorders**	
Any parent	1,975 (8.9%)
Maternal mental disorder	1,019 (4.6%)
Paternal mental disorder	1,190 (5.4%)
**Other child-onset disorders**	
Any parent	245 (1.1%)
Maternal mental disorder	126 (0.6%)
Paternal mental disorder	124 (0.6%)
**Other adult-onset disorders**	
Any parent	2,826 (12.8%)
Maternal mental disorder	1,792 (8.1%)
Paternal mental disorder	1,325 (6.0%)
**Covariates**	
Aboriginal Torres Strait Islander (Indigenous) background	1,607 (7.3%)
Socio-economic disadvantage	4,055 (18.3%)
Sex (male)	11,151 (50.4%)

**Table 2:** Distribution of parental mental disorder diagnoses among children in each schizotypy group

Any Parent Diagnosis	Total number of children with at least one affected parent	Childhood schizotypy group membership			
		*No risk*	*Introverted Schizotypy*	*Affective Schizotypy*	*True Schizotypy*
Schizophrenia-spectrum disorder	464	213 (1.8%)	110 (2.5%)	101 (2.4%)	40 (3.0%)
Affective psychotic disorder	398	172 (1.4%)	87 (1.9%)	95 (2.2%)	44 (3.3%)
Common mental disorder	3,593	1,671 (13.8%)	794 (17.8%)	814 (19.1%)	314 (24.7%)
Personality disorder	487	196 (1.6%)	118 (2.6%)	110 (2.6%)	63 (4.8%)
Substance use disorder	1,975	809 (6.7%)	453 (10.1%)	489 (11.5%)	224 (16.9%)
Other child-onset disorder	245	103 (0.9%)	56 (1.3%)	57 (1.3%)	29 (2.2%)
Other adult-onset disorder	2,826	1,251 (10.4%)	645 (14.4%)	659 (15.5%)	271 (20.5%)
No Diagnosis	16,938	9,682 (80.1%)	3,325 (74.3%)	3,084 (72.4%)	847 (64.0%)
**Total n children in each group**		12,080	4,473	4,261	1,323

Percentages in brackets are a proportion of total number of children in each schizotypy group (as represented in the bottom row). Parental diagnosis groupings are not mutually exclusive, hence columns total to more than total numbers in each schizotypy group and percentages total to over 100

### Association between schizotypy profiles and parental mental disorder

#### Any mental disorder diagnosis

Children exposed to any parental mental disorder (in at least one parent) were at greater odds of membership in each of the three schizotypy risk groups, compared to the *no risk* group, in both unadjusted (Table 3) and adjusted models (Table 4). Children with a parent with any mental disorder diagnosis had the highest odds of being in the *true schizotypy* group, followed by slightly smaller, but increased odds of being in the *introverted schizotypy* or *affective schizotypy* groups compared to the *no risk* group. Associations for any maternal or paternal mental disorder diagnosis were examined separately; the odds of schizotypy group membership were generally not substantially different for offspring of affected mothers compared to offspring of affected fathers (i.e. point estimates were similar in size and confidence intervals overlapped), with the exception of the *affective schizotypy* group which was more strongly associated with paternal (than maternal) affective psychoses and common mental disorders in both unadjusted and adjusted analyses, and with paternal personality disorders in adjusted analyses only. Children who had a history of *both* parents being diagnosed with a mental disorder had the highest odds of being in any of the schizotypy groups (compared to just one parent diagnosed), with medium sized associations with the *introverted schizotypy* and *affective schizotypy* groups, and large associations with the *true schizotypy* group.

**Table 3:** Unadjusted associations between parental mental disorder diagnosis and schizotypy group membership (*n* = 22,137)

	Schizotypy group membership		
	Introverted Schizotypy	Affective Schizotypy	True Schizotypy
	uOR (95% CI)	uOR (95% CI)	uOR (95% CI)
*Any diagnosis*			
Any parent	1.39 (1.29–1.51)	1.54 (1.42—1.67)	2.27 (2.01–2.56)
Maternal mental disorder	1.44 (1.32–1.58)	1.45 (1.32—1.59)	2.16 (1.89–2.47)
Paternal mental disorder	1.38 (1.24–1.55)	1.69 (1.51–1.88)	2.24 (2.00–2.73)
Both parents	1.82 (1.51–2.19)	1.92 (1.60–2.31)	3.04 (2.38–3.89)
*Schizophrenia-spectrum disorder*			
Any parent	1.41 (1.11–1.77)	1.35 (1.06—1.72)	1.74 (1.23–2.45)
Maternal mental disorder	1.58 (1.17–2.13)	1.26 (0.91–1.76)	1.81 (1.15–2.84)
Paternal mental disorder	1.21 (0.85–1.72)	1.41 (1.01–1.99)	1.88 (1.16–3.05)
*Affective psychotic disorder*			
Any parent	1.37 (1.06–1.78)	1.58 (1.23–2.03)	2.38 (1.70–3.33)
Maternal mental disorder	1.29 (0.97–1.72)	1.28 (0.96–1.71)	2.24 (1.55–3.24)
Paternal mental disorder	1.68 (1.05–2.69)	2.78 (1.83–4.20)	2.50 (1.32–4.73)
*Common mental disorder*			
Any parent	1.34 (1.23–1.48)	1.47 (1.34–1.61)	1.94 (1.69–2.22)
Maternal mental disorder	1.31 (1.18–1.46)	1.35 (1.22–1.50)	1.80 (1.54–2.10)
Paternal mental disorder	1.42 (1.23–1.65)	1.78 (1.54–2.05)	2.20 (1.79–2.71)
*Personality disorder*			
Any parent	1.64 (1.30–2.07)	1.61 (1.27–2.04)	3.03 (2.27–4.05)
Maternal mental disorder	1.70 (1.28–2.25)	1.40 (1.03–1.90)	3.14 (2.21–4.45)
Paternal mental disorder	1.77 (1.21–2.58)	2.11 (1.47–3.04)	3.03 (1.87–4.91)
*Substance use disorders*			
Any parent	1.57 (1.39–1.77)	1.81 (1.61–2.03)	2.84 (2.42–3.33)
Maternal mental disorder	1.78 (1.52–2.09)	1.71 (1.45–2.01)	3.23 (2.63–3.97)
Paternal mental disorder	1.53 (1.31–1.79)	1.88 (1.62–2.18)	2.80 (2.29–3.43)
*Other child-onset disorder*			
Any parent	1.47 (1.06–2.05)	1.58 (1.14–2.18)	2.61 (1.72–3.95)
Maternal mental disorder	1.35 (0.86–2.13)	1.32 (0.83–2.10)	2.63 (1.50–4.60)
Paternal mental disorder	1.64 (1.03–2.62)	2.03 (1.30–3.18)	3.27 (1.85–5.79)
*Other adult-onset disorder*			
Any parent	1.46 (1.32–1.62)	1.58 (1.43–1.75)	2.23 (1.93–2.58)
Maternal mental disorder	1.54 (1.36–1.75)	1.51 (1.33–1.71)	2.25 (1.89–2.68)
Paternal mental disorder	1.46 (1.26–1.69)	1.72 (1.50–1.98)	2.22 (1.82–2.72)

Reference group = ‘no risk’. Paternal mental disorder (and both parent) analyses sample size *n* = 21,476

*uOR =* unadjusted odds ratio, *CI* = confidence interval

**Table 4:** Associations adjusted for sex, Indigenous background, and socio-economic disadvantage between parental mental disorder diagnosis and schizotypy group membership (*n* = 22,137)

	Schizotypy group membership		
	Introverted Schizotypy	Affective Schizotypy	True Schizotypy
	aOR (95% CI)	aOR (95% CI)	aOR (95% CI)
*Any diagnosis*			
Any parent	1.27 (1.67–1.38)	1.46 (1.34–1.59)	2.04 (1.79–2.32)
Maternal mental disorder	1.29 (1.17–1.43)	1.35 (1.23–1.49)	1.88 (1.63–2.17)
Paternal mental disorder	1.27 (1.14–1.43)	1.58 (1.41–1.76)	2.03 (1.73–2.39)
Both parents	1.58 (1.30–1.91)	1.72 (1.42–2.08)	2.38 (1.85–3.07)
*Schizophrenia-spectrum disorder*			
Any parent	1.26 (1.00–1.60)	1.23 (0.96–1.56)	1.42 (1.00–2.01)
Maternal mental disorder	1.43 (1.05–1.94)	1.15 (0.82–1.60)	1.49 (0.94–2.36)
Paternal mental disorder	1.09 (0.76–1.56)	1.27 (0.90–1.79)	1.50 (0.92–2.45)
*Affective psychotic disorder*			
Any parent	1.29 (0.99–1.68)	1.49 (1.16–1.93)	2.12 (1.51–2.98)
Maternal mental disorder	1.22 (0.91–1.63)	1.21 (0.90–1.62)	2.01 (1.38–2.92)
Paternal mental disorder	1.55 (0.96–2.50)	2.61 (1.72–3.96)	2.15 (1.13–4.11)
*Common mental disorder*			
Any parent	1.27 (1.16–1.40)	1.41 (1.29–1.55)	1.77 (1.54–2.03)
Maternal mental disorder	1.24 (1.11–1.38)	1.30 (1.17–1.45)	1.64 (1.40–1.92)
Paternal mental disorder	1.34 (1.16–1.56)	1.69 (1.46–1.95)	1.97 (1.60–2.43)
*Personality disorder*			
Any parent	1.48 (1.17–1.88)	1.46 (1.15–1.86)	2.54 (1.89–3.42)
Maternal mental disorder	1.57 (1.18–2.09)	1.29 (0.95–1.75)	2.69 (1.88–3.85)
Paternal mental disorder	1.55 (1.05–2.27)	1.89 (1.31–2.72)	2.37 (1.45–3.88)
*Substance use disorder*			
Any parent	1.40 (1.24–1.59)	1.67 (1.48–1.88)	2.41 (2.04–2.85)
Maternal mental disorder	1.55 (1.31–1.83)	1.53 (1.29–1.81)	2.59 (2.09–3.21)
Paternal mental disorder	1.39 (1.18–1.63)	1.73 (1.49–2.01)	2.35 (1.92–2.91)
*Other child-onset disorder*			
Any parent	1.27 (0.91–1.77)	1.44 (1.04–2.00)	2.07 (1.36–3.17)
Maternal mental disorder	1.16 (0.73–1.84)	1.23 (0.77–1.97)	2.14 (1.21–3.77)
Paternal mental disorder	1.44 (0.89–2.32)	1.83 (1.17–2.87)	2.57 (1.44–4.59)
*Other adult-onset disorder*			
Any parent	1.34 (1.21–1.49)	1.48 (1.34–1.64)	1.94 (1.67–2.26)
Maternal mental disorder	1.42 (1.25–1.61)	1.41 (1.24–1.60)	1.95 (1.63–2.33)
Paternal mental disorder	1.33 (1.15–1.55)	1.58 (1.37–1.83)	1.87 (1.52–2.31)

Reference group = ‘no risk’. Paternal mental disorder (and both parent) analyses sample size n = 21,476

*aOR* = adjusted odds ratio, *CI =* confidence interval

#### Broad diagnostic categories

All seven categories of parental mental disorder diagnoses were associated with increased odds of offspring membership in each of the three schizotypy risk groups, in both unadjusted and adjusted analyses. For every category of mental disorder, the largest odds were for membership in the *true schizotypy* group (with medium to large effect sizes), relative to the odds ratios for membership in the *introverted *and *affective schizotypy* groups (small to medium sized effects). In unadjusted analyses, associations were generally in the small to medium sized range for most types of parental mental disorder diagnosis with the exception of personality disorders, substance use disorders and other child-onset disorders, which had large effect sizes in relation to child membership in the *true schizotypy* group. As for the analyses of the ‘any parental mental disorder’ index, there were not substantial differences in size of associations between maternal and paternal mental disorders when analyses were conducted separately. When analyses were adjusted for child sex, Indigenous background, and socio-economic disadvantage, the size of associations attenuated slightly, but most remained significant. Exceptions were the associations between any parental diagnosis of schizophrenia-spectrum disorder and offspring *affective schizotypy*, and any parental diagnosis of affective psychotic disorder or child-onset disorder with offspring *introverted schizotypy*, which were no longer significant after adjustment for demographic indicators.

## Discussion

In a large Australian population-based cohort, children with at least one parent diagnosed with a mental disorder were more likely to self-report particular combinations of cognitive, behavioural and unusual perceptual experiences that placed them in one of three schizotypal risk profiles at age 11 years, relative to children of parents without a history of mental disorder diagnosis. There was some evidence of differential relationships between familial liability to mental disorder and the three distinct subgroups of schizotypy, with parental mental illness being more strongly associated with membership of the *true schizotypy* group, relative to the likelihood of being classed in the *introverted schizotypy* or *affective schizotypy* groups. However, no particular diagnostic category of parental mental disorder was specifically associated with any subtype of schizotypy in offspring.

Children of parents with mental disorder diagnoses were two- to threefold more likely to be classed in the *true schizotypy* group compared to children of unaffected parents, while children with affected parents had only a small increase in odds of being classified into either the *introverted* or *affective schizotypy* groups. These findings are consistent with previous research indicating that the negative (e.g. interpersonal) features of schizotypy are more strongly related to familial liability for schizophrenia-spectrum disorders than positive (i.e. reality distortion) features [32], given that the *true schizotypy* group was distinguished by a pattern of high cognitive disorganisation, impulsive non-conformity, introversion, and self-other disturbance, while the *affective schizotypy* group was characterised by high unusual experiences and affective symptoms.

The association between parental mental disorder diagnosis and offspring schizotypy was consistent across a broad range of parental mental disorder diagnoses, including psychotic, affective, personality, and substance use disorders, indicating that offspring schizotypy was not specifically related to any one parental mental disorder diagnostic category. This is consistent with previous findings suggesting that intergenerational transmission of mental illness is generally non-specific [19–21, 41] and that specific parental mental disorder are associated not just with offspring mental disorder diagnosis, but also a broad spectrum of childhood vulnerability or liability to psychopathology [22], suggesting intergenerational transmission of mental illness does not follow systems of diagnostic categorisation. Interestingly, children of parents with personality disorders were most likely to be classified in any of the schizotypy groups, followed by children of parents with substance use disorders (with medium to large sized associations). While the personality disorder group did include cluster A disorders (i.e. schizotypal personality disorder, schizoid personality disorder, and paranoid personality disorder), only a very small number of individuals in the population had received these diagnoses (*n* = 15), so it is unlikely that these specific personality disorders were driving the relationship with offspring schizotypy.

When analyses were conducted separately for maternal and paternal mental disorders, there were largely no differences in the strength of relationships between parental mental disorder diagnosis and offspring schizotypy. The exception was that the *affective schizotypy* group was more strongly associated with several paternal mental disorders (affective psychoses, common mental disorders, and personality disorders) than the equivalent maternal disorders. As girls are overrepresented in the *affective schizotypy* group, this may be consistent with previous research indicating opposite-sex-specific parent-of-origin effects in the intergenerational transmission of psychosis [42]. Children with both parents diagnosed with any mental disorder (not necessarily the same disorder), were at a higher risk of being classed in any of the three schizotypy groups, relative to the likelihood of offspring schizotypy among children with only one parent with a diagnosis. This is consistent with previous findings that having two parents with mental disorders is associated with greater risk of offspring psychopathology than having one affected parent [9, 19], and suggests a dose–response relationship between parental mental disorders and offspring schizotypy and that are not necessarily concordant.

Contrary to our hypotheses, children of parents with schizophrenia-spectrum disorders were not the most likely to be represented in the three schizotypy risk profiles, but in fact had amongst the lowest odds of membership in any of the schizotypal risk profiles. This adds to previous findings that PLEs in offspring are associated with parental mental disorders other than schizophrenia-spectrum disorders [28–30], by showing that this pattern occurs not just for children with high levels of PLEs or unusual experiences (i.e. the *affective schizotypy* group), but also for children with high levels negative and disorganised symptoms but without high levels of PLEs (i.e. the *true* and *introverted* schizotypy groups). While schizotypy has generally been assumed to represent liability for schizophrenia-spectrum disorders, there is limited research examining the relationship of schizotypy with other non-psychotic mental health outcomes, and it is possible that schizotypy represents liability for a broader range of expressions of mental illness. In addition to evidence for the transdiagnostic intergenerational transmission of mental disorders [19–21, 41], multiple mental disorder diagnoses within an individual are common, and can co-occur both simultaneously and across the lifespan [43, 44]. It is therefore likely that liability for psychopathology is largely general rather than specific to certain diagnostic categories [23, 45]. Notably, schizotypy was measured in middle childhood (age ~ 11 years) in this study and may therefore be distinct from schizotypal characteristics proximal to psychosis onset that may be more specific predictors of schizophrenia-spectrum disorders.

Findings that any type of parental mental disorder diagnosis is related to offspring schizotypy are consistent with a model of transdiagnostic intergenerational transmission of mental disorder that likely operates via both genetic and environmental factors [45, 46]. Without access to genetic data for this population, we were unable to determine the extent to which genetic versus environmental factors (or an interaction or correlation between the two) account for the relationships between parental mental disorders and offspring schizotypy reported here. Potential genetic effects likely operate both directly and indirectly via environmental factors; that is, genetically influenced traits of the parents (including those that contribute to parental mental disorders) would no doubt influence the environment that offspring are raised in, which may increase the risk of schizotypy [47]. Child maltreatment has been shown to mediate a significant proportion of the association between an individual’s schizophrenia polygenic risk score and PLE’s in young adults [48]. Other environmental factors that also potentially interact with genetic vulnerability in this way include prenatal and neighbourhood factors that have also been associated with schizotypy [49]. It is thus likely that intergenerational transmission of schizotypy results from the complex interplay of multiple genetic factors and multiple environmental factors [26].

Strengths of the study include the large sample size, which allowed us to examine a broad range of parental mental disorders including relatively rare disorders (e.g. schizophrenia-spectrum disorders, personality disorders), and avoided the sampling bias associated with a high-risk sampling approach. Further, the person-centred analysis approach (i.e. latent profile analysis) allowed us to delineate groups of people with distinct patterns of schizotypal traits, rather than examining individual dimensions of schizotypy in a way that ignores population or sample heterogeneity. However, the main limitation of this study is that parental mental health diagnoses were derived from health (outpatient, inpatient, and emergency) department records only, such that parents who received care from private mental health or primary care practitioners, or who were unable to access mental health services would be classified as unexposed. Our findings may therefore underestimate the strength of associations. Further, any mental disorder diagnoses recorded before the period for which health data were available (approximately four years prior to the children’s birth) would not have been captured in the available records, potentially leading to the misclassification of children as not exposed to parental mental disorders. Second, data on presentations for parent mental diagnoses could have been recorded up to a year *after* offspring schizotypy was measured. These data were included since only the dates of diagnosis (and not the date of symptom onset) were available. It was therefore assumed that these records would largely relate to mental disorders present before offspring schizotypy was measured. Although the reverse association (i.e. offspring schizotypy causing parental mental disorder) is unlikely, this cannot be ruled out. Finally, schizotypy was measured by self-report questionnaire, which is typically less effective than clinical interview at distinguishing relatives of people with schizophrenia from controls [50]. Children may have limited insight into their own symptoms, and limited ability to assess whether symptoms are culturally normative [51]. However, self-report is valuable in that it can measure psychological content that may not be directly observable.

Clarifying the familial link between a broad spectrum of parental mental disorders and offspring schizotypy in childhood is important for understanding intergenerational pathways to adult mental disorder. The present results indicate that schizotypy in middle childhood is not specific to offspring of parents with schizophrenia-spectrum disorders, but that schizotypy is more common among offspring of parents with any type of mental disorder diagnosis. Evidence of differential relationships of familial liability to mental disorder among the distinct schizotypy risk profiles in the child population further suggests that there may be graded liability for mental health risk among the offspring of parents with mental disorders. Future research of childhood schizotypy as a potential mediator of familial liability to severe mental illness is warranted in genetically informed, intergenerational population samples.

### Supplementary Information

Below is the link to the electronic supplementary material.

Supplementary file1 (DOCX 26 KB)

## Data Availability

The linked administrative data used in this study is owned by the Australian Government and cannot be made available to third parties by the authors.
