# Supplementary material for: Parental mental disorders and offspring schizotypy in middle childhood: an intergenerational record linkage study
Source: Soc Psychiatry Psychiatr Epidemiol. 2023 Mar 13;58(11):1637–48. doi: 10.1007/s00127-023-02455-7 (PMC10562332; doi:10.1007/s00127-023-02455-7)
Supplement: Supplementary file 1 — Supplementary file1 (DOCX 26 KB) [file 127_2023_2455_MOESM1_ESM.docx]

**Parental mental disorders and offspring schizotypy in childhood: an intergenerational record linkage study**

SUPPLEMENTARY MATERIAL

**Authors**

Kirstie O’Hare^1^, Kristin R. Laurens^1,2^, Stacy Tzoumakis^1,3^, Kimberlie Dean^1,4^, Felicity Harris^1^, Vaughan J. Carr^1,5,6^, Melissa J. Green^1,5^

**Author Affiliations**

^1^ Discipline of Psychiatry and Mental Health, University of New South Wales, Sydney, AUSTRALIA

^2^ Queensland University of Technology (QUT), School of Psychology and Counselling, Brisbane, AUSTRALIA

^3^School of Criminology and Criminal Justice, Griffith University, Southport, AUSTRALIA

^4^Justice Health and Forensic Mental Health Network, Sydney, New South Wales, AUSTRALIA

^5^Neuroscience Research Australia, Sydney, AUSTRALIA

^6^ Department of Psychiatry, Monash University, Melbourne, AUSTRALIA

**Table of Contents**

[Supplementary Table 1. Descriptive statistics and psychometric properties for raw scores for the six schizotypy domains used in the latent profile analysis in the NSW-CDS (n=22,137). 2](#_Toc95918736)

[Supplementary Figure 1. Four profile solution from the latent profile analysis of six schizotypy domains 3](#_Toc95918737)

[Supplementary Table 2. Broad parental mental illness diagnostic category definitions and associated ICD-10 codes 4](#_Toc95918738)

# Supplementary Table 1. Descriptive statistics and psychometric properties for raw scores for the six schizotypy domains used in the latent profile analysis in the NSW-CDS (n=22,137).

|  |  | **n items** | **Range** | ***M*** | ***SD*** | **α** | **AIC** |
| --- | --- | --- | --- | --- | --- | --- | --- |
|  | Schizotypy Domains |  |  |  |  |  |  |
| 1 | Unusual Experiences | 12 | 0-24 | 9.86 | 5.06 | 0.83 | 0.35 |
| 2 | Cognitive Disorganisation | 6 | 0-12 | 4.33 | 2.67 | 0.73 | 0.43 |
| 3 | Impulsive Non-conformity | 14 | 0-28 | 7.86 | 4.89 | 0.82 | 0.30 |
| 4 | Introversion (Asociality) | 9 | 0-18 | 3.47 | 2.82 | 0.77 | 0.22 |
| 5 | Anxiety and Depression | 6 | 0-12 | 3.33 | 2.75 | 0.78 | 0.43 |
| 6 | Self-Other Disturbance | 12 | 0-24 | 4.90 | 4.09 | 0.84 | 0.30 |

*Note.* α = Cronbach’s alpha; AIC = Average inter-item correlation.

# Supplementary Figure 1. Four profile solution from the latent profile analysis of six schizotypy domains

# Supplementary Table 2. Broad parental mental illness diagnostic category definitions and associated ICD-10 codes

| ***Broad Diagnostic Group*** | ***Diagnostic categories*** | ***ICD-10 codes*** |
| --- | --- | --- |
| **Schizophrenia-Spectrum Disorders** | Schizophrenia | F20, F20#, F20.0, F20.1, F20.2, F20.3, F20.4, F20.5, F20.6, F20.8, F20.81, F20.89, F20.9, F23.1, F23.11, F23.2, F23.20, F23.21, F23.30, F23.31, F23.80, F23.81, F23.90, F23.91 |
|  | Schizoaffective disorders | F25, F25.0, F25.1, F25.2, F25.8, F25.9 |
|  | Other non-affective psychoses | F22, F22.0, F22.8, F22.9, F23, F23.0, F23.01, F23.3, F23.31, F23.8, F23.80, F23.9, F23.90, F23.91, F24, F28, F29, F23PC |
| **Affective Psychotic Disorders** | Bipolar disorders | F30, F30.0, F30.1, F30.8, F30.9, F30.10, F30.11, F31, F31.0, F31.1, F31.10, F31#, F31PC, F31.3, F31.30, F31.31, F31.4, F31.6, F31.7, F31.8, F31.9, F31.60, F31.64, F31.70, F31.74, F31.76, F31.81 |
|  | Postnatal affective psychoses | F53.1 |
|  | Depressive psychosis | F32.3, F32.30, F32.31, F33.3 |
|  | Other affective psychoses | F30.2, F31.2, F31.5, F32.3, F32.30, F32.31, F33.3 |
| **Common Mental Disorders** | Depressive disorders | F32, F32.0, F32.00, F32.01, F32.1, F32.11, F32.2, F32.20, F32.21, F33.2, F32.8, F32.80, F32.81, F32.89, F32.9, F32.90, F32.91, F32.10, F32#, F33, F33.0, F33.1, F33.4, F33.8, F33.9, F34, F34.0, , F34.1, F34.8, F34.9, F38, F38.0, F38.1, F38.8, F39, F53.0 |
|  | Major depressive disorders | F32.2, F32.20, F32.21, F33.2 |
|  | Anxiety and neurotic disorders | F F40, F40.0, F40.00, F40.01, F40.1, F40.2, F40.8, F40.9, F40.0PC, F40.10, F40.2, F40.210, F40.248, F41, F41.0, F41.1, F41.2, F41.3, F41.8, F41.9, F41.0P, F41.2P, F41.0PC, F41.2PC, F42, F42.0, F42.1, F42.2, F42.8, F42.9, F43, F43.0, F43.1, F43.12, F43.2, F43.20, F43.21, F43.22, F43.23, F43.24, F43.25, F43.29, F43.8, F43.9, F44, F44.0, F44.1, F44.4, F44.5, F44.6, F44.7, F44.8, F44.81, F44.88, F44.89, F44.9, F45, F45.0, F45.1, F45.2, F45.3, F45.30, F45.32, F45.33, F45.34, F45.38, F45.4, F45.41, F45.42, F45.8, F45.9, F45PC, F48, F48.0, F48.1, F48.8, F48.9 |
| **Personality Disorders** | Cluster A | F21, F60.0, F60.1 |
|  | Cluster B | F60.2, F60.3, F60.30, F60.31, F60.4 |
|  | Cluster C | F60.5, F60.6, F60.7 |
|  | Other Personality Disorders | F60, F60.8, F60.09, F60.9, F61, F62, F62.0, F62.1, F62.8, F62.9, F68.0, F68.1, F68.8, F69 |
| **Substance Use Disorders** | Substance Intoxication | F10, F10.0, F11, F11.0, F12, F12.0, F13, F13.0, F13.00, F14, F14.0, F15, F15.0, F15.00, F16, F16.0, F16.00, F17, F17.0, F18, F18.0, F19, F19.0 |
|  | Substance Use Disorders | F10.1, F10.10, F10.19, F10.2, F10.121, F10.129, F10.20, F10.21, F10.220, F10.229, F10.230, F10.231, F10.239, F10.3, F10.4, F10.6, F10.8, F10.9, F10.94, F10.950, F10.951, F10.96, F10.980, F10.988, F10PC, F11.1, F11.10, F11.129, F11.19, F11.2, F11.20, F11.23, F11.3, F11.4, F11.8, F11.9, F11#, F12.1, F12.10, F12.20, F12.921, F12.2, F12.3, F12.4, F12.7, F12.8, F12.9, F13.1, F13.09, F13.10, F13.11, F13.19, F13.2, F13.129, F13.20, F13.21, F13.29, F13.230, F13.3, F13.30, F13.31, F13.39, F13.49, F13.96, F13.980, F13.90, F13.99, F13.4, F13.40, F13.41, F14.1, F14.10, F14.2, F14.20, F14.23, F14.3, F14.4, F14.8, F14.9, F14.90, F14.929, F15.01, F15.02, F15.09, F15.1, F15.10, F15.11, F15.12, F15.129, F15.19, F15.9, F15.2, F15.20, F15.21, F15.22, F15.29, F15.3, F15.30, F15.31,F15.39, F15.4, F15.41, F15.42, F15.49, F15.52, F15.6, F15.8, F15.80, F15.81, F15.89, F15.90, F15.91, F15.92, F15.921, F15.93, F15.94, F15.980, F15.99, F16.1, F16.10, F16.19, F16.2, F16.20, F16.3, F16.30, F16.4, F16.50, F16.9, F16.90, F17.1, F17.2, F17.203, F17.210, F17.3, F17.4, F17.9, F18.1, F18.10, F18.2, F18.3, F18.4, F18.9, F19.1, F19.10, F19.2, F19.20, F19.239, F19.3, F19.4, F19.6, F19.8, F19.9, F19.929, F19.939, F19.950, F19.959, F55.0, F55.1, F55.2, F55.5, F55.8, F55.9 |
| **Other Adult Onset Disorders** | Other postnatal conditions | F53, F53.8, F53.9 |
|  | Other Disorders not elsewhere classified | F99, F99.1 |
|  | Other Adult onset disorders (e.g. organic disorders, eating disorders, sleep disorders) | F00, F00.0, F00.1, F00.2, F00.9, F00#, F01, F01.0, F01.1, F01.2, F01.3, F01.8, F01.9, F02.3, F02.8, F03, F04, F04.0, F048, F04.00, F04.01, F04.02, F04.03, F05, F05.0, F05.1, F05.8, F05.9, F06, F06.0, F06.1, F06.2, F06.3, F06.30, F06.32, F06.33, F06.34, F06.4, F06.5, F06.6, F06.7, F06.8, F06.9, F07, F07.0, F07.1, F07.2, F07.8, F07.81, F07.9, F45.20, F45.29, F09, F50, F50.0, F50.00, F50.01, F50.1, F50.2, F50.3, F50.4, F50.5, F50.8, F50.89, F50.9, F51, F51PC, F51.0, F51.01, F51.1, F51.2, F51.3, F51.4, F51.5, F51.8, F51.9, F52, F52.0, F52.1, F52.2, F52.3, F52.4, F52.5, F52.6, F52.7, F52.8, F52.9, F52PC, F54, F55, F58.9, F59, F63, F63.0, F63.1, F63.2, F63.3, F63.8, F63.89, F63.9,F64, F64.0, F64.1, F64.2, F64.8, F64.9, F65, F65.0, F65.1, F65.2, F65.3, F65.4, F65.5, F65.6, F65.8, F65.9, F66, F66.0, F66.1, F66.2, F66.8, F66.9 |
|  | Self-harm | R45.81, X84 |
|  | Mental health diagnosis not yet allocated | 99.1 |
| **Other Childhood Onset Disorders** | Hyperkinetic disorders | F90, F90.0, F90.1, F90.8, F90.9 |
|  | Conduct Disorders | F91, F91.0, F91.1, F91.2, F91.3, F91.8, F91.9, F92, F92.0, F92.8, F92.9, F91#, F92# |
|  | Other childhood emotional disorders | F93, F93.0, F93.1, F93.2, F93.3, F93.8, F93.9 |
